# Measuring perceived self-location in virtual reality

**DOI:** 10.1038/s41598-020-63643-y

**Published:** 2020-04-22

**Authors:** Estelle Nakul, Nicolas Orlando-Dessaints, Bigna Lenggenhager, Christophe Lopez

**Affiliations:** 1 Aix Marseille Univ, CNRS, LNSC, FR3C Marseille, France; 2 Department of Psychology, University of Zurich, Zurich, Switzerland

**Keywords:** Cognitive neuroscience, Navigation

## Abstract

Third-person perspective full-body illusions (3PP-FBI) enable the manipulation, through multisensory stimulation, of perceived self-location. Perceived self-location is classically measured by a locomotion task. Yet, as locomotion modulates various sensory signals, we developed in immersive virtual reality a measure of self-location without locomotion. Tactile stimulation was applied on the back of twenty-five participants and displayed synchronously or asynchronously on an avatar’s back seen from behind. Participants completed the locomotion task and a novel mental imagery task, in which they self-located in relation to a virtual ball approaching them. Participants self-identified with the avatar more during synchronous than asynchronous visuo-tactile stimulation in both tasks. This was accentuated for the mental imagery task, showing a larger self-relocation toward the avatar, together with higher reports of presence, bi-location and disembodiment in the synchronous condition only for the mental imagery task. In conclusion, the results suggest that avoiding multisensory updating during walking, and using a perceptual rather than a motor task, can improve measures of illusory self-location.

## Introduction

Self-location, i.e. the experience that the self occupies a certain volume of space, is considered a core aspect of bodily self-consciousness, together with self-identification and first-person perspective^1–3^. Self-location is typically experienced within the physical limits of the body^1,4^, but can be experienced as disembodied in various neurological and psychiatric conditions^5^. Self-location has proven difficult to study empirically. Early measures of self-location consisted of introspective reports^6–8^, and pointing tasks on human silhouettes or toward the participant’s body^9,10^. They revealed that most participants located their self in the head or torso. Self-reports of self-location have also been collected during illusions with sets of mirrors^11,12^ and video systems^13^. These studies indicate the possibility to manipulate self-location through unusual visuo-spatial perspectives, in a way that the perceived self-location deviates from the location of the physical body.

Whole-body adaptations of the rubber hand illusion^14^ accelerated the empirical study of the multisensory foundations of self-location, especially with the development of full-body illusions (FBI) from a third-person perspective (here referred to as 3PP-FBI)^15–19^. 3PP-FBIs are characterized by self-identification with a full body in extrapersonal space rather than with a body part in peripersonal space. In a seminal version of the 3PP-FBI, participants wore a head-mounted display (HMD) in which they observed a video of their body or a mannequin’s body filmed from behind and projected to the front^18^. Tactile stimulation was applied on the participants’ back and the video was either shown in synchrony or with a delay relative to the stimulation, so that participants saw and felt the touch on the back synchronously or asynchronously. Results show that the integration of spatially dissociated, but synchronous, visual and tactile events increased self-identification with the virtual body. During such self-identification the participant’s skin temperature was found to decrease^20^.

To date, very few studies have used immersive virtual reality (VR) technology to implement 3PP-FBIs^21–23^. Most studies of self-location and self-identification were based on pre-recorded or online video-projections of bodies^16,18,24–28^. Yet, the study of self-location should benefit from VR. First, VR allows interacting with avatars in realistic, ecological, and controlled environments^29–32^. Second, VR is characterized by *presence*, the feeling of being “there”, even when a virtual character is not shown in the VR environment, modifying the perceived self-location^30,33^.

How can self-location be measured in VR, other than with questionnaires? In most 3PP-FBI studies self-location was measured by a locomotion task (LT), an action-based (motor) judgement in which participants were moved backward and asked to walk to where they perceived to be located during the visuo-tactile stimulation^15,18,34^. Participants relocated their self from 10 to 30 cm toward the seen body from their initial position after synchronous visuo-tactile stimulation^35,36^. Thus, self-location was a compromise between the location of the physical body and the location of the seen body participants self-identified with.

An often-reported limitation of the LT is that locomotion updates somatosensory, vestibular and interoceptive signals. Measures of self-location based on perceptual judgements rather than action-based judgements could minimize self-motion, reduce confounding sensory stimulation, and maintain illusory self-location. There is evidence to suggest that action-based judgments are less sensitive to visual illusions, as they rely on different neural pathways, than more perceptual judgments^37–39^. To measure self-location in non-moving participants, a “mental ball dropping task” has been used during 3PP-FBIs using video presentations of a human body^16,17,19^. Participants tested lying supine held a ball and had to indicate, with button presses, when they imagined dropping the ball and when the ball would hit the ground. After synchronous visuo-tactile stimulation, participants’ evaluation of the time needed for the ball to reach the ground increased or decreased with regard to their illusion of being located above or below their body. Similar results were reported for a mental ball dropping task in VR^40^. However, the mental ball dropping task needs to be adapted to a more ecological 3PP-FBI in which participants are tested standing upright. Body positioning is important as lying supine modifies corporeal and extracorporeal space perception^41^. To our knowledge, there is still a lack of a measure of self-location which does not require changes in perceived self-location with respect to gravity, but rather with respect to the more ecological front-back or left-right axes. Second, participants exposed to VR are thought to distribute their self between the real and VR environments^33^, which may impact self-location. A measure of self-location limiting participants’ interactions with their real environment is likely to maintain presence and the 3PP-FBI in VR.

The present study primarily aimed to measure changes in self-location after a 3PP-FBI in a fully immersive VR environment using and comparing a perceptual (based on mental imagery) task and a locomotor task. Differences between perceptual and motor tasks have already been reported for the rubber hand illusion^42^, in line with perception-action dissociations reported for visual illusions^37,38^. Here, we adapted the mental ball dropping task to measure self-location in the anterior-posterior horizontal axis. Our new mental imagery task (MIT) aimed to measure changes in self-location while participants stand upright and still, avoiding confounding effects of sensorimotor updating through locomotion, as in previous studies^18^. Results from the MIT were compared to those of the same participants in the LT. Finally, as previous studies showed that empathy and depersonalization/derealization traits can influence bodily self-consciousness^43,44^, a secondary aim of the study was to explore their relation to the 3PP-FBI in VR.

## Methods

### Participants

Twenty-five healthy volunteers participated (11 females; mean age ± SD: 23 ± 2 years). They were all right-handed (mean laterality quotient ± SD: 89 ± 14% according to the Edinburgh Handedness inventory^45^). They had normal or corrected-to-normal vision and declared no history of neurological or psychiatric disease. All participants provided informed written consent prior to participation. Experimental procedures were approved by the local Ethics Committee (Comité de Protection des Personnes Sud-Méditerranée II) and followed the ethical recommendations laid down in the Declaration of Helsinki.

### Experimental setup

**Figure 1:**
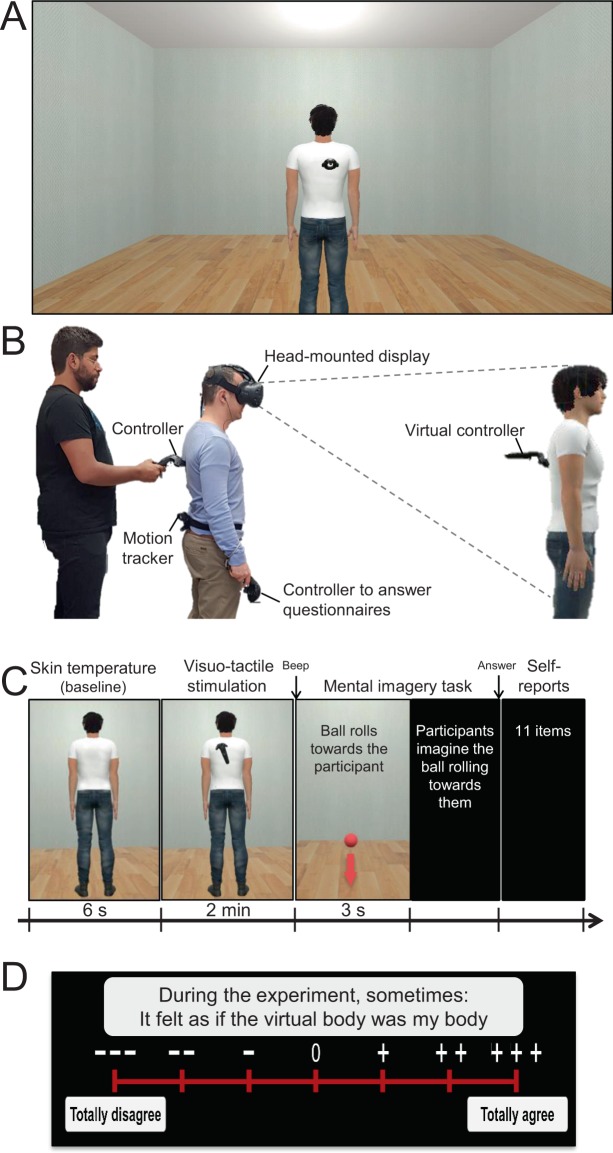
Experimental setup and procedures. (**A**) Participants were immersed through an HMD at the centre of a 7 × 4 m virtual room. An avatar was shown 2 m in front of the participants at the centre of the room. (**B**) During visuo-tactile stimulation, the experimenter stroked the back of the participants with a controller while its movement was synchronously or asynchronously reproduced by a virtual controller on the back of the avatar. Participants wore a motion tracker at the waist to record their position during the locomotion task (LT). They held a controller in their right hand to answer during the mental imagery task (MIT) and to answer the questionnaires. (**C**) A block of MIT comprised the following events: (1) Baseline measurement of the skin temperature during 6 s; (2) 2 min of synchronous or asynchronous visuo-tactile stimulation. (3) A beep indicates the onset of the MIT, followed by (4) the apparition of a red ball in front of the participants, in the end of the room, which rolled at constant speed toward “them” during 3 s. (5) A black screen appeared and participants imagined the ball rolling toward them and (6) they indicated when they thought that the ball had arrived at their position, by pulling the controller’s trigger. (7) The answer to the localization task triggered the presentation of a questionnaire. (**D)** Participants used their controller to direct a virtual laser to one of the levels of a Likert scale ranging from “Totally disagree” to “Totally agree”.

During the whole experiment, participants were immersed in a VR environment (Unity 3D, Unity Technologies) using an HMD (HTC Vive, HTC Corporation) with a horizontal visual field of 110° (~90° per eye). Participants were placed at the centre of a virtual room (7 × 4 m) of approximatively similar size and decoration to the real room, to facilitate comparisons between measures of self-location in the MIT and LT (Fig. 1A). A motion tracker was placed at the waist at the back of the participants, to record their position in space, and they held a VR controller in their right hand (Fig. 1B). Two laser-emitter and inertial units encoded 3D coordinates of the HMD, VR controllers and the tracker, which were all recorded at 90 Hz with Unity. Participants wore earphones transmitting white noise during the experiment to avoid distractions and spatial auditory information.

### Visuo-tactile stimulation

An avatar wearing a pair of jeans and a white t-shirt (created with Morph3D) was shown at the centre of the VR room, approximately 2 m in front of the participant’s viewpoint (Fig. 1A). The experimenter stroked the participants’ back with a VR controller, irregularly so that they could not predict the tactile stimulation on their back, while the controller movement was reproduced by a virtual depiction of the controller on the back of the avatar (Fig. 1B). During synchronous visuo-tactile stimulation, the movement of the virtual controller on the back of the avatar matched the movement and position of the controller on the back of the participant. During asynchronous visuo-tactile stimulation, the virtual controller reproduced the movement of the controller on the back of the participant with a 500 ms delay. Synchronous and asynchronous visuo-tactile stimulations were each applied for 2 minutes in separate blocks.

### Measures of self-location

#### Mental imagery task

After the visuo-tactile stimulation, the avatar was removed from the VR room and a beep tone played in the earphones indicated the start of the MIT. A red ball appeared in front of the participants at the far end of the VR room (Fig. 1C). The ball rolled on the floor of the VR room along an invisible line toward the participants, at constant velocity for 3 s, after which a black screen was shown. Participants were instructed to imagine the ball rolling toward them at the same velocity and to indicate when the ball would reach “them”, at a position between their feet, by pressing on a button on the VR controller. To infer participant’s self-location, we recorded the coordinates of the (hidden) ball along the anterior-posterior axis when participants responded.

#### Locomotion task

After the visuo-tactile stroking, a black screen was presented in the HMD. Following previous procedures^18^, participants walked on the spot while being guided 2 m backward by the experimenter. Then, they were instructed to walk with normal steps to the position they thought they were standing during the visuo-tactile stimulation. Participants were asked not to count the number of small steps during the backward displacement. The final position of the participant was measured from the coordinates of the tracker on their waist.

### Subjective reports

After each block, an eleven-item questionnaire presented in the HMD measured experiences during the visuo-tactile stimulation (Table 1). Questions pertained to different aspects of embodiment highlighted in previous research, such as self-identification, touch referral, disembodiment, agency, illusory movement, bi-location and presence. Participants answered on a seven-point Likert scale ranging from “Totally disagree” to “Totally agree” (Fig. 1D) to the questions presented sequentially. They used a virtual laser pointer, originating from the controller in their hand, directed the laser toward a level of the scale and validated their choice with the controller’s trigger. Answering the questionnaire in the HMD, instead of on paper sheets as usually done, enabled participants to stay in VR space and prevented interactions with the real environment that could add sensory stimulation irrelevant to the experiment.

**Table 1:** Questionnaire about experiences during the 3PP-FBI.

	Questions # – During the experiment there were times when:
*Self-identification*	Q3 – It felt as if the virtual body was my body.
	Q6 – It seemed as if I might have had more than one body.
*Touch referral*	Q1 – It seemed as if I were feeling the touch of the VR controller in the location where I saw the virtual body (hence on the virtual body).
	Q2 – It seemed as though the touch I felt was caused by the virtual controller touching the virtual body.
*Disembodiment*	Q5 – It seemed as if the touch I was feeling came from somewhere between my own body and the virtual body.
*Agency*	Q9 – It seemed as if I could animate (put in motion) the virtual body as my own body if I had wanted.
*Illusory movement*	Q4 - It felt as if my (real) body was drifting toward the front (toward the virtual body).
	Q7 - It appeared as if the virtual body were drifting backward (toward my body).
*Bi-location*	Q8 - It seemed as if I were in two places at the same time.
*Presence*	Q10 - It seemed as if I were located in the virtual scene.
*Nausea*	Q11 - I felt sick to my stomach (nausea).

Questions were presented sequentially, in the order indicated by their number. Questions pertaining to the same experience are grouped in the Table.

### Measures of depersonalization and empathy

Participants completed the 60-item Empathy Quotient (EQ^46^), gauging individual empathy traits. Ratings were completed on a four-point scale ranging from “Strongly disagree” to “Strongly agree”.

Participants also filled out the 29-item Cambridge Depersonalization Scale (CDS^47^), which assesses the frequency and duration of sensations of depersonalization and derealization. Ratings for the frequency and duration of these experiences were given on two scales ranging from 0 (“Never”) to 4 (“Always”), and from 1 (“A few seconds”) to 6 (“More than a week”), respectively.

### Temperature recordings

Body temperature was recorded at 1024 Hz using a probe placed over the sixth cervical vertebra (Biosemi Inc., Amsterdam, Netherlands).

### Session organization

1. Before the experiment proper, the avatar’s shape and size were adapted to match each participant. Participants were trained to the MIT and LT to get familiarized with instructions and procedures, and they were trained to manipulate the VR controller to answer questionnaires in VR. The temperature probe was installed before the experiment to stabilize the signal.
2. In the second phase, the procedures for the measures of self-location with the MIT and LT were explained to participants in detail. After this, we collected *pre-tests measures* of self-location without any visuo-tactile stimulation, in line with previous studies^18^. We recorded pre-test measures of self-location in one block of six trials of the MIT, and pre-test measures of self-location in one block of three trials of the LT. The number of trials was chosen on the basis of a pilot study^48^. Pre-test measures were collected only once for each task, before the experiment proper started.
3. The experimenter proper comprised four blocks of stimulation. At the beginning of each block, participants observed the avatar for 6 s before the onset of the 2-minute visuo-tactile stimulation. This allowed to record baseline body temperature. After each type of visuo-tactile stimulation, participants completed one trial of either the MIT or the LT. This resulted in four experimental blocks presented in a quasi-counterbalanced order across participants: Synchronous visuo-tactile stimulation followed by the MIT, Asynchronous visuo-tactile stimulation followed by the MIT, Synchronous visuo-tactile stimulation followed by the LT, and Asynchronous visuo-tactile stimulation followed by the LT. After each block, participants completed the eleven questions about their subjective experience during the visuo-tactile stimulation.
4. Participants filled out the EQ and CDS on paper sheets at the end of the experiment.

### Data processing and statistical analysis

#### Self-location

For both tasks, self-location was expressed in Unity metric units (UM) along the anterior–posterior axis. *Drifts *in self-location were calculated by subtracting the mean anterior-posterior coordinate of the ball (MIT), or of the tracker on the participant’s waist (LT), during the pre-tests session from the coordinate recorded after synchronous or asynchronous visuo-tactile stimulation. Thus, drifts represent the distance between mean self-location before the FBI and self-location after the blocks of visuo-tactile stimulation. Drifts were analysed using a 2 Tasks (MIT, LT) × 2 Synchrony (Synchronous, Asynchronous) repeated-measures ANOVA and by t-tests for post hoc analyses of significant main effects and interactions, which were Bonferroni corrected for multiple comparisons (SPSS Statistics 22; IBM Corp., Armonk, NY, USA). Alpha level was set to 0.05. We explored differences with p ≤ 0.06 which, although non-significant, may warrant further investigation. A posteriori power analysis for self-location measurements with an effect size of 0.25, α = 0.05 and power=0.8 indicates a theoretical sample size of n = 24 (G*Power^49^), indicating that our study had enough statistical power.

#### Subjective reports

As data were not normally distributed, the scores were first analysed using Friedman’s ANOVAs to test the effect of the Block (Synchronous MIT, Asynchronous MIT, Synchronous LT, Asynchronous LT) on each question independently. Significant effects were analysed using Wilcoxon signed-rank tests to assess the effect of the Synchrony for each Task, and the effect of the Task separately for synchronous and asynchronous stimulation. To minimize the risk of false positives, we used Bonferroni-corrected α level of p < 0.05/2 for statistical significance (p < 0.025).

#### Relation to empathy and depersonalization/derealisation

We conducted an exploratory analysis using non-parametric Spearman’s correlations to analyse relations between the EQ score, the CDS score, and the *relative drift *in self-location. *Relative drifts *were calculated as the difference between the final positions after synchronous and asynchronous visuo-tactile stimulation, thus representing the distance between self-location in the synchronous compared to asynchronous visuo-tactile stimulation.

#### Temperature recordings

The two-minute recordings during the visuo-tactile stimulation were divided in 20 time windows of 6 s (i.e., T1–T20). Temperature changes were measured by subtracting the average response during the 6 s preceding the visuo-tactile stimulation from the average response over each of the 20 time windows of 6 s after the stimulation. As data were not normally distributed, the effect of Time (T1–T20) on skin temperature was analysed for each Block separately, and the effect of the Block was analysed for each time window separately, using Friedman’s tests (Bonferroni-corrected α level).

## Results

### Self-location

**Figure 2:**
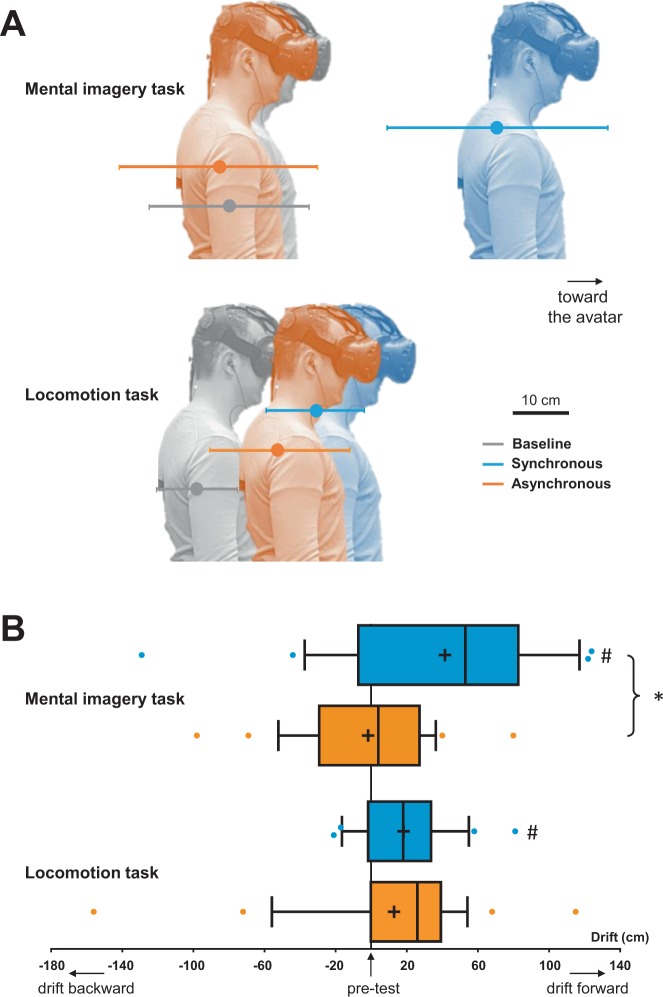
Self-location. (**A**) Mean self-location measured in the pre-test condition (grey), and after synchronous (blue) and asynchronous (yellow) visuo-tactile stimulation in the MIT and LT tasks. Errors bars represent the SEM. (**B**) Box and whiskers plots show medians and interquartile ranges (10–90) for the drifts (self-location after synchronous or asynchronous stimulation minus pre-test measure). Means are shown as + and coloured dots are outliers. * Indicates statistically significant difference between synchronous and asynchronous visuo-tactile stimulation, and # indicates statistically significant difference with respect to pre-test measures of self-location.

Figure 2A illustrates the perceived self-location for the pre-test measurement (without any stimulation), as well as for the measurements immediately after synchronous and asynchronous visuo-tactile stimulation, separately for the MIT and LT. Inspection of the data indicates larger variability in the perceptual judgement (MIT) than in the motor judgment (LT) of self-location.

To directly compare the effects of visuo-tactile stimulation on self-location in both tasks, the drifts in self-location (i.e. anterior–posterior coordinate after visuo-tactile stroking minus coordinate during pre-test measurement) were analysed (Fig. 2B). A repeated-measures ANOVA on the drifts revealed a main effect of the Synchrony (F_1,24_ = 12.85, p = 0.001, η²_p_ = 0.35, power: 0.93), but no main effect of the Task (F_1,24_ = 0,354, p = 0.558, η²_p_ = 0.02, power: 0.09). As expected, this shows that the synchrony of the visuo-tactile stimulation influenced the perceived self-location. There was also a nearly significant interaction of Synchrony × Task (F_1,24_ = 4.22, p = 0.05, η²_p_ = 0.15, power: 0.51). Exploring this interaction revealed a significantly larger drift in self-location for synchronous than for asynchronous stimulation in the MIT (t = 3.54, p = 0.002, r = 0.58). In contrast, drifts for the synchronous and asynchronous stimulation did not differ for the LT (t = 0.51, p = 0.61, r = 0.10). Finally, when directly comparing drifts from the MIT and the LT, the difference between synchronous drifts approached the α level of significance (t = 1.95, p = 0.06, r = 0.37), which was not the case for asynchronous drifts (t = −1.25, p = 0.22, r = 0.25).

Pre-tests measures were important to know whether there was a relative forward or backward displacement of the perceived self-location in synchronous and asynchronous stimulation, respectively. Figure 2B, shows that for the MIT the mean (±SEM) self-location after synchronous stimulation was 41 ± 12 cm toward the avatar, which was significantly different from zero (i.e., different from the pre-test measurement, t = 3.47, p = 0.002, r = 0.58). By contrast, the mean self-location of 2 ± 8 cm backward in the asynchronous condition was not significantly different from zero (t = −0.22, p = 0.83, r = 0.04). The LT showed a mean self-location of 18 ± 5 cm forward in the synchronous condition, which was significantly different from zero (i.e., different from the pre-test measurement, t = 3.62, p = 0.001, r = 0.59) and a mean self-location of 13 ± 10 cm toward the avatar in the asynchronous condition, which was not significantly different from zero (t = 1.26, p = 0.22, r = 0.25). We conclude that synchronous visuo-tactile stimulation displaces the perceived self-location toward the avatar both in the MIT and in the LT, whereas the asynchronous visuo-tactile stimulation has no significant effect in both tasks.

### Subjective reports

Table 2 summarizes results from the Friedman’s tests. There was a significant effect of the Block (Synchronous MIT, Asynchronous MIT, Synchronous LT, Asynchronous LT) on the intensity of self-identification (Q3, Q6), touch referral (Q1, Q2), disembodiment (Q5), agency (Q9), illusory movement (Q7), bi-location (Q8) and presence (Q10) (all χ^2^ ≥ 6.6 and p ≤ 0.02). Of note, there was no effect of the block on nausea (Q11).

**Table 2:** Self-reports. Results of the Friedman’s tests and post-hoc Wilcoxon comparisons for the significant main effects.

Questions		Friedman’s test	MIT (synch. vs asynch.)	LT (synch. vs asynch.)
*Self-identification*	Q3	χ^2^ = 37.1 p < 0.001*	Z = −3.55 p < 0.001*	Z = −3.28 p = 0.001*
	Q6	χ^2^ = 20.9 p < 0.001*	Z = −2.98 p = 0.003*	Z = −2.58 p = 0.010*
*Touch referral*	Q1	χ^2^ = 33.8 p < 0.001*	Z = −3.79 p < 0.001*	Z = −3.62 p < 0.001*
	Q2	χ^2^ = 25.5 p < 0.001*	Z = −3.65 p < 0.001*	Z = −2.08 p = 0.037
*Disembodiment*	Q5	χ^2^ = 10.2 p = 0.02*	Z = −2.43 p = 0.015*	Z = −1.99 p = 0.047
*Agency*	Q9	χ^2^ = 26.6 p < 0.001*	Z = −3.40 p = 0.001*	Z = −3.16 p = 0.002*
*Illusory movement*	Q4	χ^2^ = 6.6 p = 0.08	−	−
	Q7	χ^2^ = 11.1 p = 0.01*	Z = −2.81 p = 0.005*	Z = −1.58 p = 0.113
*Bi-location*	Q8	χ^2^ = 11.7 p = 0.01*	Z = −2.62 p = 0.009*	Z = −1.70 p = 0.089
*Presence*	Q10	χ^2^ = 9.7 p = 0.02*	Z = −2.37 p = 0.02*	Z = −1.60 p = 0.116
*Nausea*	Q11	χ^2^ = 3.5 p = 0.33	−	−

Significance is indicated by an asterisk.

**Figure 3:**
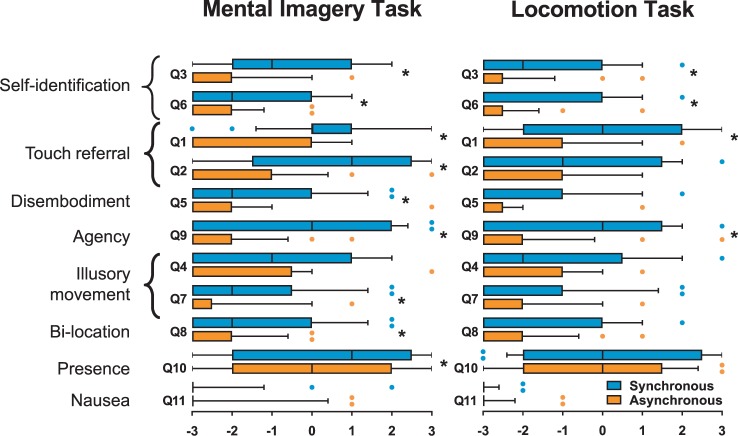
Subjective reports. Box and whiskers plots show medians and interquartile ranges (10–90) for each questionnaire item. Means are shown as + and dots are outliers. * Indicates a significant difference between synchronous and asynchronous visuo-tactile stimulation (Wilcoxon signed-rank test).

Wilcoxon tests showed that for both MIT and LT synchronous visuo-tactile stimulation induced significantly stronger feeling of self-identification (Q3; MIT: Z = −3.55, p < 0.001, r = −0.50; LT: Z = −3.28, p = 0.001, r = −0.46; Q6; MIT: Z = −2.98, p = 0.003, r = −0.42; LT: Z = −2.58, p = 0.010, r = −0.36), touch referral (Q1; MIT: Z = −3.79, p < 0.001, r = −0.54; LT: Z = −3.62, p < 0.001, r = −0.46) and agency (Q9; MIT: Z = −3.40, p = 0.001, r = −0.48; LT: Z = −3.16, p = 0.002, r = −0.45) when compared to asynchronous stimulation (Table 2 and Fig. 3).

Yet, only after the MIT did participants report stronger disembodiment (Q5; Z = −2.43, p = 0.015, r = −0.34), bi-location (Q8; Z = −2.62, p = 0.009, r = −0.37) and presence in VR (Q10; Z = −2.37, p = 0.020, r = −0.33) in the synchronous when compared to the asynchronous stimulation (Table 2 and Fig. 3). After synchronous visuo-tactile stimulation, they also reported a stronger feeling that the touch they felt was due to the virtual controller (Q2; Z = −3.65, p < 0.001, r = −0.52) and that the avatar was drifting toward them (Q7; Z = −2.81, p = 0.005, r = −0.40). No significant difference between synchronous and asynchronous stimulation was found for the LT.

When comparing the intensity of the self-reports between the MIT and LT directly, there was no overall difference. Wilcoxon tests revealed a significant difference for item Q2 only, showing higher touch referral after synchronous stimulation for the MIT compared to the LT (Z = −2.34, p = 0.019, r = − 0.33). There was no significant difference between the MIT and LT in the rating of the other questionnaire items after synchronous stimulation (all Z ≤ −1.01 and p ≥ 0.025). In addition, there were no significant differences in self-reports between the MIT and LT after asynchronous stimulation for any of the questionnaire items (all Z ≤ −0.42 and p ≥ 0.13).

### Relation to depersonalization and empathy

An exploratory analysis indicated that the relative drift in self-location after the MIT was positively correlated with EQ scores (*ρ* = 0.42, p = 0.034), meaning that more empathic participants tended to show a larger error in self-location toward the avatar after synchronous stroking. We found no correlation between the drift after the LT and EQ scores (*ρ* = 0.12, p = 0.551), nor between drifts and the CDS score. These preliminary observations should be taken with caution because of the small sample size, resulting in a lack of power for such a correlation analysis.

### Skin temperature

**Figure 4:**
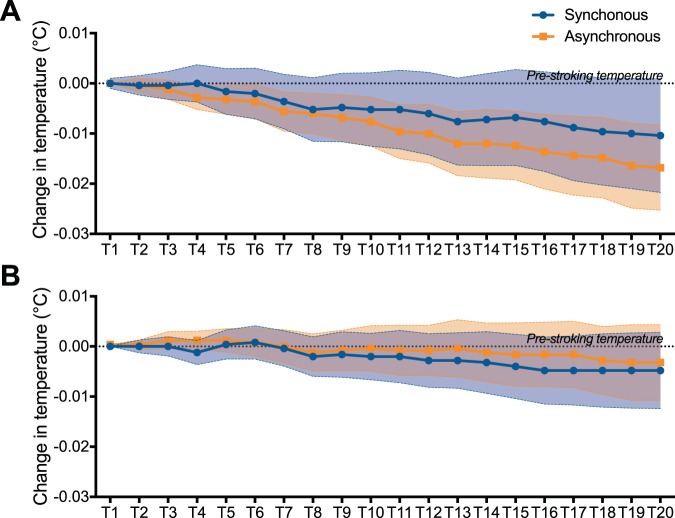
Skin temperature. Coloured dots and squares represent the mean change in skin temperature with respect to the average skin temperature during the 6 s before the onset of visuo-tactile stroking in the MIT (**A**) and LT (**B**). Colored areas represent the SEM. The horizontal black dotted line indicates the pre-stroking skin temperature (y = 0). T1 to T20 are the 20 time windows of 6 s during the synchronous and asynchronous visuo-tactile stimulation.

Figure 4 shows the change in skin temperature during the visuo-tactile stimulation. Friedman’s tests revealed a significant effect of Time (T1–T20) for the asynchronous visuo-tactile stimulation in both tasks (MIT: χ^2^ = 47.35, p < 0.001; LT: χ^2^ = 54.27, p < 0.001), indicating an overall decrease in skin temperature over time. By contrast, there was no significant effect of Time for the synchronous stimulation in both tasks (MIT: χ^2^ = 0.47, p = 1.0; LT: χ^2^ = 13.41, p = 0.82). Moreover, there was no significant effect of the experimental Block on skin temperature for time windows T1 to T20 (all χ^2^ < 3.48 and all p > 0.31). Therefore, we did not calculate correlation between skin temperature, self-location and self-reports.

## Discussion

The present study measured self-identification with an avatar and perceived self-location during a 3PP-FBI in fully immersive VR. We developed a new mental imagery task (MIT) to measure the perceived self-location in VR when participants are standing still. The MIT was compared in the same participants to a classical locomotion task (LT), in which participants walked to indicate the previously perceived self-location. As in previous studies using video-based 3PP-FBIs, in this immersive setting we found higher self-identification with a rendered standard avatar after synchronous compared to asynchronous visuo-tactile stimulation. This was also corroborated by a generally larger self-relocation toward the avatar after synchronous than asynchronous stimulation. Furthermore, the nearly significant interaction of Synchrony by Task suggested an effect of Synchrony on self-location in the MIT, but not in the LT. This was corroborated by a significant effect of Synchrony on the sense of presence in the MIT, but not in the LT. We discuss below how differences between the MIT and LT, such as stronger multisensory updating during walking (LT), or dissociation between perceptual (MIT) and action-based (LT) self-location judgements, might explain why our MIT appears more sensitive to illusory self-relocation toward an avatar.

### Virtual reality vs video-based techniques for the FBI

Our adaptation of the 3PP-FBI in fully immersive VR induced self-identification and self-relocation toward a virtual body. The main effect of Synchrony indicates that synchronous visual and tactile events contribute to the perceived self-location, and that integration of spatially mismatching signals can lead to illusory self-location and self-identification. This is in line with previous 3PP-FBI studies that used multisensory stimulation and video-based techniques^17–20^. The fact that we obtained very similar results in a VR setup regarding self-identification, in which the participants saw the touch on a standard computer-generated avatar, is relevant for both theoretical and practical reasons. On the theoretical side, it has previously been argued^50^ that a cognitive understanding of the video setup might influence the results obtained in an earlier video-based 3PP-FBI^18^. The fact that the VR setup seems to modify self-location in the MIT in similar ways than the video-based setup contradicts this assumption. On the other hand, the fact that multisensory stimulation on a digital avatar, even when it is seen from a 3PP, increases self-identification and alters self-location in the MIT demonstrates the feasibility of future VR studies. For practical reasons, VR setups enable much better experimental control than video-based setups, as both the avatar and environment can be altered easily and in real time, which enlarges possible research questions^30^.

In the present study, the LT showed no difference between self-location for synchronous and asynchronous stimulation, but only a significant difference between synchronous stimulation and pre-tests measures. Our previous video-based 3PP-FBI study also found no significant difference between synchronous and asynchronous stimulation in the LT (see Fig. 3 in Ref^18^.), but found that the synchronous stimulation differed significantly from the pre-test self-location measures. Future studies should endeavour to compare directly, in the same participants, the intensity of illusory self-location and self-identification in computer-generated and video-based setups.

### Illusory self-location and self-identification in the MIT *vs* LT

Comparing self-location and self-identification in the MIT and LT indicates that the MIT is a more appropriate measure of the strength of the 3PP-FBI when using immersion in VR. The MIT, but not the LT, revealed larger drifts after the synchronous than the asynchronous visuo-tactile stimulation. Differences between the tasks were also found at the level of self-reports, as participants reported only for the MIT stronger presence, bi-location and disembodiment during synchronous stimulation. We thus propose that the MIT, in which participants compared their position to that of a virtual ball rolling toward “them”, is more appropriate to measure self-location after an FBI in immersive VR.

We propose that the main advantage of the MIT is to restrict sensorimotor updating as participants stood upright and motionless (apart from their natural postural oscillations) throughout the experiment and task. By contrast, the larger change in vestibular, somatosensory and visceral graviceptor signals, and thus larger multisensory updating in the LT may lead to an “anchoring” of the self to the body. This is supported by a large body of evidence from research on postural control showing that whole-body interactions with the environment, especially the forces developed through the dynamics of balance, favour more accurate vertical and space perception than do still and motionless body postures^51,52^. Along the same line of argument, out-of-body experiences occur more often in lying or sitting positions than in standing upright position or during whole-body motion^5^. Two sensory systems seem especially important in driving these effects: the vestibular system and proprioception. Experiments in healthy participants showed that vestibular stimulation can influence the degree of anchoring of the self to the body as well as self-identification in variants of the FBI^53–55^. Similarly, vibration of the ankles (that stimulate muscle spindles from the legs), but not vibration of the wrist, decreased self-identification with the avatar in a 3PP-FBI^56^.

In addition to their degree of sensorimotor updating, a key difference between both tasks is that the MIT involves a *perceptual* judgement (mental imagery of a ball), whereas the LT involves more a *motor*, *action-based *judgement of self-location (walking toward a previous self-location). Functional dissociations between perceptual and action-based judgements of a stimulus are well documented, especially for visual illusions^37,38,57^. For example, in the Müller-Lyer illusion, participants are more sensitive to the illusion when asked to compare the length of visual lines than when asked to grasp objects at the end of visual lines. As in the present study, participants walking (LT) toward targets were more accurate than when they had to compare visual distances (perceptual task)^39^.Thus, in line with earlier studies on perception-action dissociations^37–39,57^, our results suggest that the MIT, which does not involve any action, proved more sensitive than the motor task (LT) to illusory drift of self-location toward the avatar.

A limitation of the present study was that we measured self-location as one *point* in space. Although self-location has been defined as a *volume* the self occupies in space^1^, reducing self-location to a single point is convenient for laboratory measure^18^. Recent studies have postulated that drifts in self-location may reflect an expansion of the peripersonal space boundaries toward the avatar^58^. Our paradigm does not allow to disambiguate between an expansion of peripersonal space, an expansion of the volume occupied by the self, bi-location (simultaneous or alternating self-location between the real body and the avatar), or relocation of the self between the real body and the avatar. A ball rolling toward the participant was shown during the MIT, thus representing a looming visual stimulus, as was coincidentally showed in paradigms measuring peripersonal space during the FBI. Although our MIT and paradigms measuring peripersonal space are very different, we cannot exclude that the observation of a virtual ball in the MIT may have contributed to make the MIT more sensitive to the FBI.

### Presence and bi-location

An important finding of the self-report analysis was that presence and bi-location were experienced only after the MIT. Presence relies on a certain detachment from sensory information from the real environment, in favour of sensory information transmitted by the VR system^30^. Contrary to the MIT, the LT requires participants to navigate in their real environment, so that they may shift attention from the virtual sensory stimuli to their bodily signals. This may involve a shift from an allocentric reference frame in VR (whereby vision trumps the vestibular and somatosensory systems) to a more egocentric frame of reference during the LT, which was done in darkness. The bi-location reported here is consistent with previous reports in the VR literature, showing that self-location distributes progressively between the physical and the virtual spaces during immersion in VR^33^. It is also reminiscent of full-blown bi-location in neurological conditions, whereby the self is experienced both in the physical body and in a duplicate body perceived in the extrapersonal space^59^. Again, the effects on presence and bi-location found for the MIT, but not the LT, might be related to differences in sensorimotor updating between the tasks. However, shorter presentation of the black screen in the MIT than in the LT (as participants completed the MIT quicker than the LT), or visual presentation of a virtual ball rolling toward the participants (3 more seconds of VR exposure) in one task but not the other, may have also contributed to these effects.

### Influence of empathy

Our exploratory analysis suggests that empathy positively correlated with the drift in self-location after the MIT, but not after the LT. Although this result should be taken with caution because of the limited sample size and low statistical power, it suggests that more empathic participants tend to show larger mislocalisation toward the avatar after synchronous stroking. This preliminary finding is in line with the growing literature suggesting bidirectional interactions between social and own-body space^60^. Studies have shown that how strongly we empathize with others depends on, and conversely influences, the plasticity of our body and peripersonal space^44,61^. For example, shared multisensory stimulation between two participants can reduce the perceived distance between them and enlarge their peripersonal space^62^.

In the present study, empathy may have a stronger impact on self-location after the MIT, probably due to the lack of sensorimotor updating, to perception-action dissociations, and/or to the other differences between the tasks mentioned above. This again indicates that the MIT is a better measure of the perceived self-location. It is, to our knowledge, the first study suggesting that empathy correlates with self-location toward an avatar participants self-identified with. Our observation in healthy participants is congruent with results from a study in autistic population – characterized by low empathy– showing absent self-identification and illusory self-location toward the avatar in a video-based version of the 3PP-FBI. We note that the current neuroscientific models of self-consciousness have often overlooked the effects of empathy on the multisensory foundations of bodily self-consciousness. This should now be the focus of more systematic studies in more highly powered studies using larger samples of healthy participants to explore our preliminary report.

### Body temperature

We found no significant effect of visuo-tactile stimulation on body temperature, which has been suggested as an objective measure of self-identification. This null finding is in contrast to a previous 3PP-FBI study^20^. The relation of illusory self-identification with the decrease in body temperature is, however, controversial as other studies failed to replicate this finding^43,63^. Although Salomon *et al*.^20^ found temperature drops on the participants back and legs, Macauda *et al*.^54^ found an effect of the FBI on the hand, but not on the neck temperature, and they proposed that the temperature of visible body parts (the hand but not the neck in their study) can be modulated. As the avatar’s neck was visible in our study, this suggests that vision of the body part is not enough to change the skin temperature.

## Conclusion

In conclusion, we showed that self-identification and self-location in a 3PP-FBI using immersive VR depend on the spatiotemporal congruency between visual and tactile signals and that self-location correlates with empathy. Yet, the intensity of illusory self-identification and self-location also depends on the measure of self-location. Our MIT is a more appropriate measure of the strength of the 3PP-FBI when using immersion in VR. Developing mental imagery tasks such as that proposed here should help improve the study of self-location in participants standing in immersive VR environments.

## Data Availability

Data reported in this manuscript are available on request from the authors.
